# Tumoral emboli and encircling lymphovasculogenesis

**Published:** 2010-05-31

**Authors:** Sanford H. Barsky

**Affiliations:** University of Nevada and Nevada Cancer Institute, Las Vegas , NV

**Keywords:** Mahooti et al. Breast carcinomatous tumoral emboli can result from encircling lymphovasculogenesis rather than lymphovascular invasion. Oncotarget, 2010, in press.

Indirect and circumstantial clinical evidence suggests that metastasis often occurs early in tumor progression. In early stage breast cancer, for example, a significant percentage of patients exhibit circulating tumor cells (CTC's) and disseminated bone marrow tumor cells (DTC's) [[1, [2]. More direct experimental evidence for "early metastasis" has been observed in studies of transgenic mice expressing a cancer-causing oncogene where the primary cancer early on exists in an "in situ" stage yet bone marrow metastases are discovered [3]. If metastasis indeed occurs early, then the rate-limiting steps which give rise to metastasis must also occur early. Some rate-limiting steps include both angiogenesis as well as lymphangiogenesis. However there have been alternate short-circuiting mechanisms proposed for these phenomenon including vasculogenic mimicry [4]. Another key rate-limiting step in the metastatic process is tumoral embolic formation within lymphovascular channels. The canonical view of the origin of tumor lymphovascular emboli is that they directly originate from lymphovascular invasion. We found evidence, however that tumoral lymphovascular emboli can result from the short-circuiting step of encircling lymphovasculogenesis [5]. Experimentally, we used a xenograft of human inflammatory breast cancer (MARY-X), a model that exhibited florid tumor emboli, to generate tumoral spheroids *in vitro*. In observational studies, we chose human breast carcinoma cases where there appeared to be a possible transition of *in situ* carcinoma to lymphovascular emboli without intervening stromal invasion. These cases were studied by morphometry as well as IHC with tumor proliferation (Ki-67) and adhesion (E-cadherin) markers, myoepithelial (p63), as well as endothelial (podoplanin [D2-40], CD31, VEGFR-3, Prox-1) markers. Unlabelled spheroids coinjected with  either GFP or RFP-human myoepithelial cells or murine embryonal fibroblasts (MEFs) gave rise to tumors which exhibited GFP/RFP immunoreactivity within the cells lining the emboli-containing lymphovascular channels. *In vitro* studies demonstrated that the tumoral spheroids induced endothelial differentiation of cocultured myoepithelial cells and MEFs, measured by real time PCR and immunofluorescence. In humans, the DCIS clusters exhibited similar proliferation, E-cadherin immunoreactivity and size as the tumor emboli (p =.5), suggesting the possibility that the latter originated from the former. The DCIS clusters exhibited a loss (50%-100%) of p63 myoepithelial immunoreactivity but not E-cadherin epithelial immunoreactivity. The tumor emboli were mainly present within lymphatic channels whose dual p63/CD31, p63/D2-40 and p63/VEGFR-3 and overall weak patterns of D2-40/CD31/VEGFR-3 immunoreactivities suggested that they represented immature and newly created vasculature derived from originally myoepithelial-lined ducts. Collectively both experimental as well as observational studies suggested the possibility that these breast cancer emboli resulted from encircling lymphovasculogenesis rather than conventional lymphovascular invasion. Encircling lymphovasculo-genesis as the mechanism of lymphovascular invasion represents a paradigm shift in our thinking about this phenomenon. The lymphovascular embolus represents the phenomenon of the "ship in the bottle" where the arduous and complex task of constructing a ship within a bottle is replaced with the relatively effortless and simple job of building the bottle around the ship [5].
